# Postnatal Development of Numbers and Mean Sizes of Pancreatic Islets and Beta-Cells in Healthy Mice and GIPR^dn^ Transgenic Diabetic Mice

**DOI:** 10.1371/journal.pone.0022814

**Published:** 2011-07-26

**Authors:** Nadja Herbach, Martina Bergmayr, Burkhard Göke, Eckhard Wolf, Ruediger Wanke

**Affiliations:** 1 Institute of Veterinary Pathology, Center for Clinical Veterinary Medicine, LMU Munich, Germany; 2 Department of Internal Medicine II, University Clinic Grosshadern, LMU Munich, Germany; 3 Chair for Molecular Animal Breeding and Biotechnology, and Laboratory for Functional Genome Analysis (LAFUGA), Gene Center, LMU Munich, Germany; University of Ulster, United Kingdom

## Abstract

The aim of this study was to examine postnatal islet and beta-cell expansion in healthy female control mice and its disturbances in diabetic GIPR^dn^ transgenic mice, which exhibit an early reduction of beta-cell mass. Pancreata of female control and GIPR^dn^ transgenic mice, aged 10, 45, 90 and 180 days were examined, using state-of-the-art quantitative-stereological methods. Total islet and beta-cell volumes, as well as their absolute numbers increased significantly until 90 days in control mice, and remained stable thereafter. The mean islet volumes of controls also increased slightly but significantly between 10 and 45 days of age, and then remained stable until 180 days. The total volume of isolated beta-cells, an indicator of islet neogenesis, and the number of proliferating (BrdU-positive) islet cells were highest in 10-day-old controls and declined significantly between 10 and 45 days. In GIPR^dn^ transgenic mice, the numbers of islets and beta-cells were significantly reduced from 10 days of age onwards vs. controls, and no postnatal expansion of total islet and beta-cell volumes occurred due to a reduction in islet neogenesis whereas early islet-cell proliferation and apoptosis were unchanged as compared to control mice. Insulin secretion in response to pharmacological doses of GIP was preserved in GIPR^dn^ transgenic mice, and serum insulin to pancreatic insulin content in response to GLP-1 and arginine was significantly higher in GIPR^dn^ transgenic mice vs. controls. We could show that the increase in islet number is mainly responsible for expansion of islet and beta-cell mass in healthy control mice. GIPR^dn^ transgenic mice show a disturbed expansion of the endocrine pancreas, due to perturbed islet neogenesis.

## Introduction

The functional pancreatic beta-cell mass is critical for adequate insulin secretion, and therefore maintenance of glucose homeostasis. Beta-cell mass is dynamic throughout life and may adapt to meet the actual demand [1]. It is generally believed that neogenesis is the major driving process in the perinatal and in the early postnatal period to rapidly expand beta-cell mass, whereas self-replication is thought to contribute mainly to the slow expansion of islet and beta-cell mass in later stages of postnatal pancreas development under physiological conditions [2]. There are some conflicting data in literature about the dynamics of numbers and mean volumes of islets and beta-cells, but it seems generally believed that islet number is fixed in postnatal life, whereas mean islet size is thought to increase (islet hypertrophy) with age or increasing demand [2], [3], [4], [5]. Beta-cell mass is thought to increase via both beta-cell hyperplasia and hypertrophy [1]. Changes in islet size may be important considering functionality (cell-cell interactions, blood supply) [5], [6]. The aims of this study were to analyze the postnatal development of total numbers and mean volumes of islets and beta-cells of female control mice (CD1 outbred genetic background), and to determine the morphological features of disrupted postnatal beta-cell mass expansion of diabetic mice, expressing a dominant negative glucose-dependent insulinotropic polypeptide receptor (GIPR^dn^), using unbiased quantitative-stereological methods.

GIP is an incretin hormone, which is released into the blood stream from enteroendocrine cells after food intake, and produces multiple physiological effects, including enhancement of glucose-mediated insulin secretion and insulin gene transcription, and may act as a mitotic and anti-apoptotic factor in pancreatic beta-cells [7], [8], [9], [10], [11].

Previously, we have described a transgenic diabetic mouse model, expressing a dominant negative GIP receptor in pancreatic beta cells [12]. The cDNA of the human GIPR was mutated at the third intracellular loop (deletion of amino acids 319–326, Ala→Glu exchange at position 340). The loss of function of the mutated GIPR was demonstrated *in vitro*. Transgenic mice were then generated, expressing the mutated human *GIPR* cDNA under the control of the rat pro-insulin 2 gene promoter in pancreatic beta cells. These GIPR^dn^ transgenic mice exhibit an early disturbance in pancreatic islet development (severe reduction of beta-cell mass, disturbed composition of islets with alpha-, delta- and PP-cell hyperplasia and decreased islet neogenesis), diminished insulin secretion, hyperglucagonemia and early onset diabetes mellitus. The reduction in beta-cell mass precedes development of hyperglycemia and is therefore thought to result from transgene expression [12].

## Materials and Methods

### Animals

All animal experiments were performed according to the German Animal Welfare Act with permission of the responsible animal welfare authority (Regierung von Oberbayern, approval ID 55.2-1-54-2531.2-26-06 and 94-07). Transgenic mice were generated as previously described [12]. Transgenic males were bred onto a CD1 background (Charles River, Germany). DNA was extracted from tail tips as previously described, and transgenic mice were identified by polymerase chain reaction [12]. Since our previous investigations [12] revealed no difference in the development of total islet and beta-cell volumes between male and female control mice and since the phenotype of GIPR^dn^ transgenic mice is not influenced by gender, one gender (female) was chosen for analyses.

Mice were maintained on a 12-h light and 12-h dark cycle and received a standard breeding diet (C1314, Altromin, Germany) and tap water *ad libitum*.

### In vivo investigations

Blood was withdrawn from randomly fed mice at 10 days of age, and at 45, 90 and 180 days of age after a 15-hour fasting period, and following 1.5 hours re-feeding. Blood glucose was measured, using the Super GL_easy_ System (Dr. Müller Gerätebau, Germany), serum insulin and malondialdehyde were determined, using the sensitive Rat Insulin RIA kit (Linco Research, USA) or the TBARS Assay kit (Cayman Chemical Company, USA), respectively.

At the age of 50 days, an insulin sensitivity test was carried out with randomly fed mice. At the beginning, a basal blood sample was taken from the nicked tail tip to determine blood glucose (t = 0 minutes). Thereafter, 1U insulin /kg body weight was injected intraperitoneally. Further blood samples were obtained at 10, 20, 30 and 60 minutes for determination of blood glucose (Super GL_easy_ System), and the relative decrease from basal blood glucose levels was determined.

Glucose, GIP, and GLP-1 stimulation tests were performed at 10 days of age with randomly fed animals as described previously [13], using 1.5 mg/kg body weight glucose and 1.5 mg/kg glucose with either 1.7 mg/kg GIP or GLP-1 (Bachem, Germany). Arginine stimulation tests were performed at 10 days of age, using 1 mg/g body weight arginine in 0.9% NaCl. Animals were euthanized 10 min after application of secretagogues or randomly fed (basal values) by bleeding from the retroorbital plexus under anesthesia, the pancreas was removed and insulin was extracted using 0.18 M HCl in 95% alcohol over night. Blood glucose was determined as described above, serum insulin and pancreatic insulin content was determined, using the Ultra Sensitive Mouse Insulin ELISA kit (Chrystal Chem Inc, USA). The increase in insulin secretion from basal was calculated dividing insulin concentrations after stimulation by mean basal levels (fold insulin secretion) or by the pancreatic insulin content (relative secretion).

### Isolated islets

Pancreatic islets were isolated at 10 days of age after neutral red perfusion via the left heart ventricle and digestion of the pancreas with collagenase Type I (Sigma, Germany) by handpicking under a stereomicroscope. For determination of the cAMP content, batches of 10–15 islets were boiled in 0.05 M HCl for 3 min, dried by vacuum centrifugation (Vacuum concentrator, Bachofer, Germany), resuspended in working buffer (Biomedical Technologies Inc., USA) and subjected to cAMP EIA (Biomedical Technologies Inc.).

### Immunohistochemistry

The indirect immunoperoxidase method was used to determine insulin containing cells as described previously [12], as well as BrdU-positive (Oxford Biotechnology, Great Britain), and apoptotic cells (TUNEL, MP Biomedicals, France) on paraffin embedded pancreatic tissue. Horseradish-conjugated rabbit anti-guinea pig IgG, and rabbit anti-rat IgG were from DAKO Diagnostika (Germany). The horseradish peroxidase labeled streptavidin biotin method was used to localize insulin containing cells in plastic embedded tissue, using the same guinea pig anti-insulin antibody, but diluted 1∶50 in phosphate buffered saline (PBS). A biotinylated pig anti-rabbit IgG (1∶50, Dako), which is known to cross-react with guinea pig Ig, was used as secondary antibody.

### Quantitative-stereological investigations of the pancreas

Blood was collected from the tail vein to determine fasting blood glucose levels and 1 ml/100 g body weight of a 10 mM BrdU solution was injected intraperitoneally. Pancreas preparation was performed as described previously [12], [14] with some modifications. After fixation in 10% neutral buffered formalin, the pancreas was embedded in agar, sectioned perpendicular to its longitudinal axis into parallel slices of approximately 1 mm thickness, with the first cut positioned randomly within an interval of 1 mm length at the splenic end of the pancreas. Slices were placed in tissue capsules on a piece of foam-rubber sponge with the right cut surface facing downward. Then embedding in paraffin (every first slice) and plastic (GMA/MMA; every second slice) was performed. The morphological changes of the endocrine pancreas were evaluated quantitatively applying unbiased model-independent stereological methods [15] as described [12], [14].

### Total volumes of islets, beta-cells, and isolated beta-cells

Total volumes of islets, beta-cells and isolated beta-cells - an indicator for islet neogenesis [16], [17] - were determined essentially as described previously [14], using the following equations:

1) Pancreas volume:

V_Pan_  =  Pancreas weight/specific weight of mouse pancreas (1.08 mg/mm^3^)

2) Total islet volume:

V_(Islets,Pan)  = _ ∑A_islets_/∑A_pancreas_ x V_Pan_

3) Total beta-cell volume:

V_(B-cells,islets)  = _ ∑A_B-cells_/∑A_islets_ x V_(Islets,Pan)_

4) Total volume of isolated beta-cells:

V_(Neo,Pan)  = _ ∑A_isolated beta-cells_/∑A_pancreas_ x V_Pan_

∑A equals the sum of cross-sectional areas of the respective compartment.

**Figure 1:**
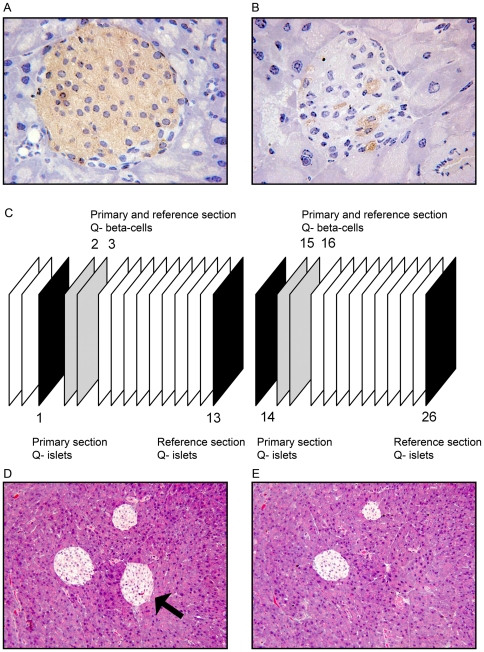
Representative islet profiles, sampling of sections, counting Q^−^ islets. Example of an islet profile of a control (A) and a GIPR^dn^ transgenic mouse (B) immunohistochemically stained for insulin; (C) Sampling scheme for drawing primary and reference sections; (D) Primary section and (E) reference section for counting Q^−^ islets, one Q^−^ may be counted in the example (arrow in E).

An example of anti-insulin stained islet profiles of a control and a transgenic mouse is shown in Fig. 1 A and B.

### Determination of number and size of islets and beta-cells

Total islet and total beta-cell numbers were determined using plastic sections, applying the disector method [18]. The physical disector, which consists of a pair of physical section planes separated by a known distance, was used to estimate the numerical density of islets in the pancreas and of beta-cells in the islets. The distance between both sections (disector height) was chosen to be approximately 30% of the mean minimum diameter of the objects to be counted [19], and accounted for 18 µm and 1.5 µm for islets and beta-cells, respectively. The mean minimum diameter of islets and beta-cells was determined planimetrically. The plastic embedded pancreas was cut exhaustively into 1.5 µm thick sections, and the primary sections for counting islets were sampled beginning at a random start between the first and 12^th^ section, the corresponding reference section was sampled 12 sections ahead (Fig. 1C). All sampled sections for determination of islet numbers were stained with H&E. For counting Q^−^ beta-cells, three series of adjacent sections were selected from the sections not used for H&E-staining. The primary section was chosen, beginning at a random start between section 2 and 11, the reference section was drawn one section ahead (Fig. 1C). These selected plastic sections were stained immunohistochemically for insulin.

A particle (Q^−^) is counted when it is present in the primary but not in the reference section. The process of counting Q^−^ was then repeated by interchanging the roles of the primary and reference section, thereby increasing the efficiency by a factor of two.

Q^−^ islets were counted, using two microscopes (final magnification 100x). Clusters of endocrine cells were only regarded as islets when at least five nuclear profiles were visible. A section pair for counting Q^−^ islets is illustrated in Fig. 1D/E. Following equations were used for calculations:

1) Numerical volume density of islets in the pancreas:

Nv_(Islets/Pan)_  =  Q^−^/(∑A_pancreas_ x h) x fs^3^

2) Number of islets in the pancreas

N_(Islets,Pan)_  =  Nv_(Islets/Pan)_ x V_(Pan)_

3) Mean islet volume


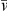
_(Islets)_  =  ∑A_islets_/∑A_pancreas_ / Nv_(Islets/Pan)_

h: disector height (18 µm), fs: linear tissue shrinkage factor (0.91±0.02); on the average 40 Q^−^ were counted per animal.

For counting Q^−^ beta-cells, immunohistochemically stained sections were used: with a random start within the first slice of pancreas of the primary section, islets in every second strip were sampled until a total number of five islets in wild-type mice and seven islets in transgenic mice was reached. Photographs of the selected islets and the corresponding islets in the reference section were taken, and printed at a final magnification of 1530x. A plastic transparency with an unbiased counting frame was applied to the print-outs, and the Q^−^ beta-cells were counted according to the unbiased counting rule [18]. The area of the counting frame corresponds to the disector area (∑A_disector_). In case of small islets which were smaller than the counting frame, the whole islet area was used as disector area.

The following equations were used for calculations:

4) Numerical volume density of beta-cells in islets

Nv_(B-cells/Islets)_  =  Q^−^/(∑A_disector_ x h) x fs^3^

5) Total number of beta-cells in the islets

N_(B-cells,Islets)_  =  Nv_(B-cells/Islets)_ x V_(B-cells, Islets)_.

6) Mean beta-cell volume


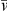
_(B-cells)_  =  ∑A_B-cells_/∑A_islets_ / Nv_(B-cells/Islets)_.

h: disector height (1.5 µm), fs: linear tissue shrinkage factor (0.91±0.02); On the average, 47 Q^−^ were counted per animal.

### Islet-cell replication and apoptosis

BrdU and TUNEL positive nuclear profiles of islet-cells were counted, using immunohistochemically stained paraffin sections.

With a random start within a section of the first slice of pancreas, the whole slide was surveyed by meandering, neglecting every second strip. At 10 days of age, all islet profiles were analyzed. Stained and un-stained nuclear profiles of islet-cells were counted and the areas of the corresponding islets were measured planimetrically. The total number of islet cell nuclei was obtained as the product of the numerical area density of islet cell nuclei and the islet area. The numbers of BrdU and TUNEL positive nuclear profiles are stated as number of positive cells per 100,000 nuclei.

### Statistical analyses

Statistical analyses of all data but insulin tolerance tests were performed, using one-way analysis of variance (ANOVA), differences between genetic groups and age-groups were determined by pair wise comparisons, using the LSD test, with *P*<0.05 indicating statistical significance (IBM SPSS Statistics 19.0, IBM Deutschland GmbH, Germany). Data from insulin tolerance tests were compared by Mann Whitney U-test. Quantitative stereological data were logarithmically transformed to approximate normal distribution. Data are presented as means and standard error of means (SEM) throughout the study.

## Results

### Postnatal development of islets and beta-cells in healthy CD1 mice

**Figure 2:**
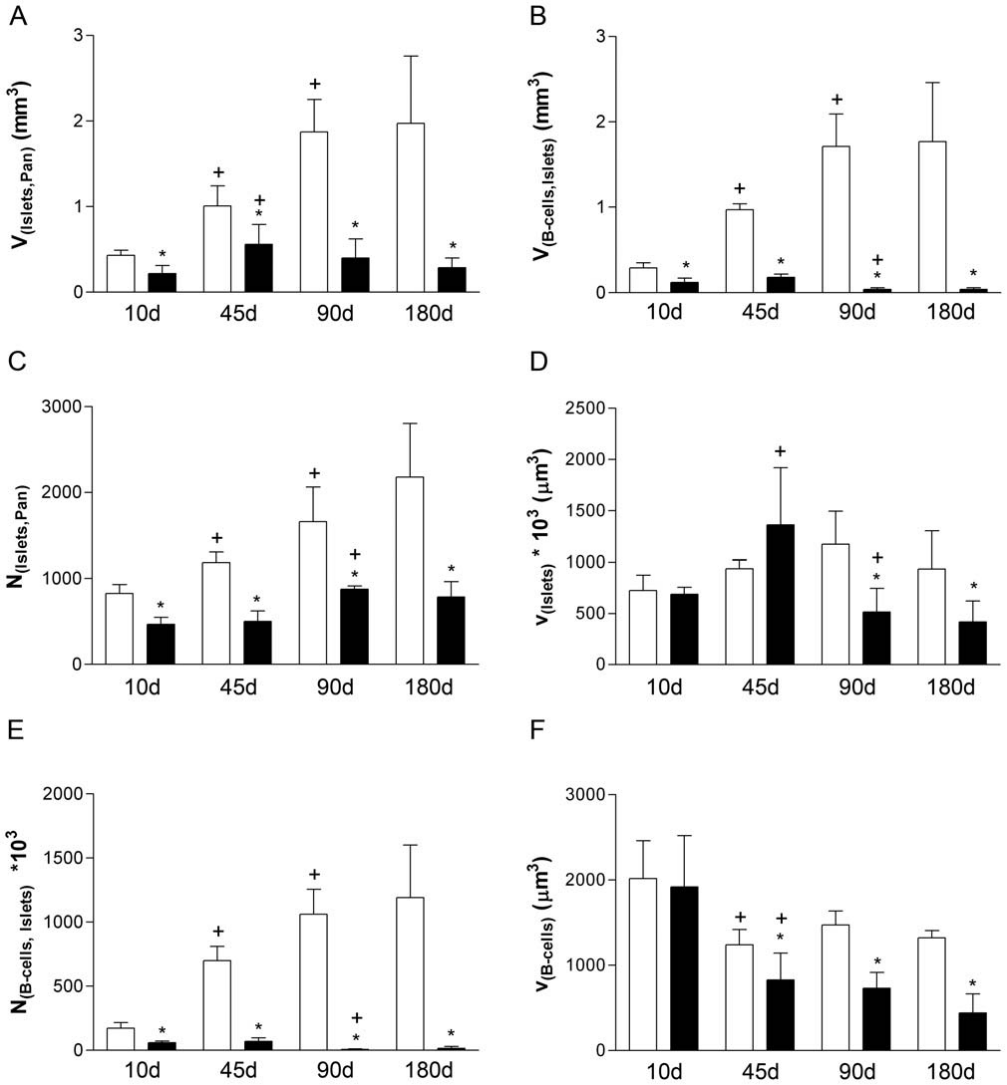
Quantitative-stereological investigations of the endocrine pancreas. (A) Total islet volume (V_(Islets,Pan)_, (B) total beta-cell volume (V_(B-cells,Islets)_, (C) Number of islets (N_(Islets, Pan)_, (D) mean islet volume (v_(islets)_, (E) beta-cell number (N_(B-cells,Islets)_, (F) mean beta-cell volume (v_(B-cells)._ Open bars: female control mice; filled bars: female GIPR^dn^ transgenic mice; Data represent means and SEM. * p<0.05 vs. age-matched control; +p<0.05 vs. previous time point.

The total islet and beta-cell volumes more than tripled in healthy control mice between 10 and 90 days of age and remained stable thereafter (Fig. 2A, B). This increase in islet and beta-cell mass was associated with a two-fold increase in islet number at 90 vs. 10 days of age and islet number was 2.6-fold higher at 180 vs. 10 days of age (Fig. 2C). The mean islet volume of controls did not change significantly in the period investigated (Fig. 2D). The total number of beta-cells in the pancreas was 7-times higher at 180 days vs. 10 days of age (Fig. 2E), whereas the mean beta-cell volume decreased significantly between 10 and 45 days of age (by 39%) and remained stable thereafter (Fig. 2F).

**Figure 3:**
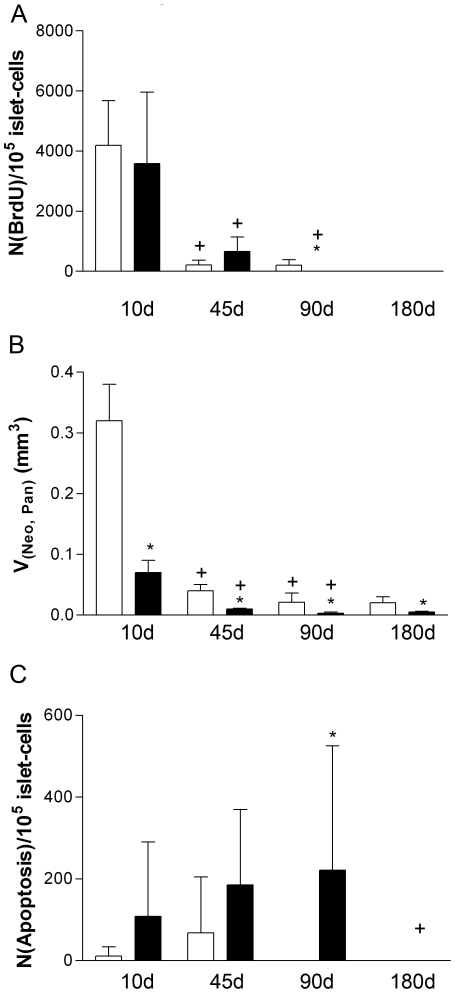
Islet-cell replication, isolated beta-cells, and islet-cell apoptosis. (A) number of BrdU positive islet cells per 10^5^ cells (N_(BrdU)_), (B) total volume of isolated beta-cells (V_(Neo,Pan)_, (C) number of apoptotic islet cells per 10^5^ cells (N_(Apoptosis)_). Open bars: female control mice; filled bars: female GIPR^dn^ transgenic mice; Data represent means and SEM. * p<0.05 vs. age-matched control; +p<0.05 vs. previous time point.

The increase in both total islet and beta-cell volumes and numbers was associated with high islet-cell replication and neogenesis of islets at 10 days of age. Islet-cell replication declined between 10 and 45 days of age, and was undetectable in 180-day-old control females (Fig. 3A). The total volume of isolated beta-cells, an indicator for islet neogenesis [16], [17], declined significantly between 10 and 45 days of age, and remained at low levels thereafter (Fig. 3B). Low levels of islet-cell apoptosis were detectable at 10 and 45 days of age, no apoptosis was demonstrable in controls thereafter (Fig. 3C).

### Impaired glucose tolerance, reduced insulin levels, oxidative stress and insulin resistance in GIPR^dn^ transgenic mice

**Figure 4:**
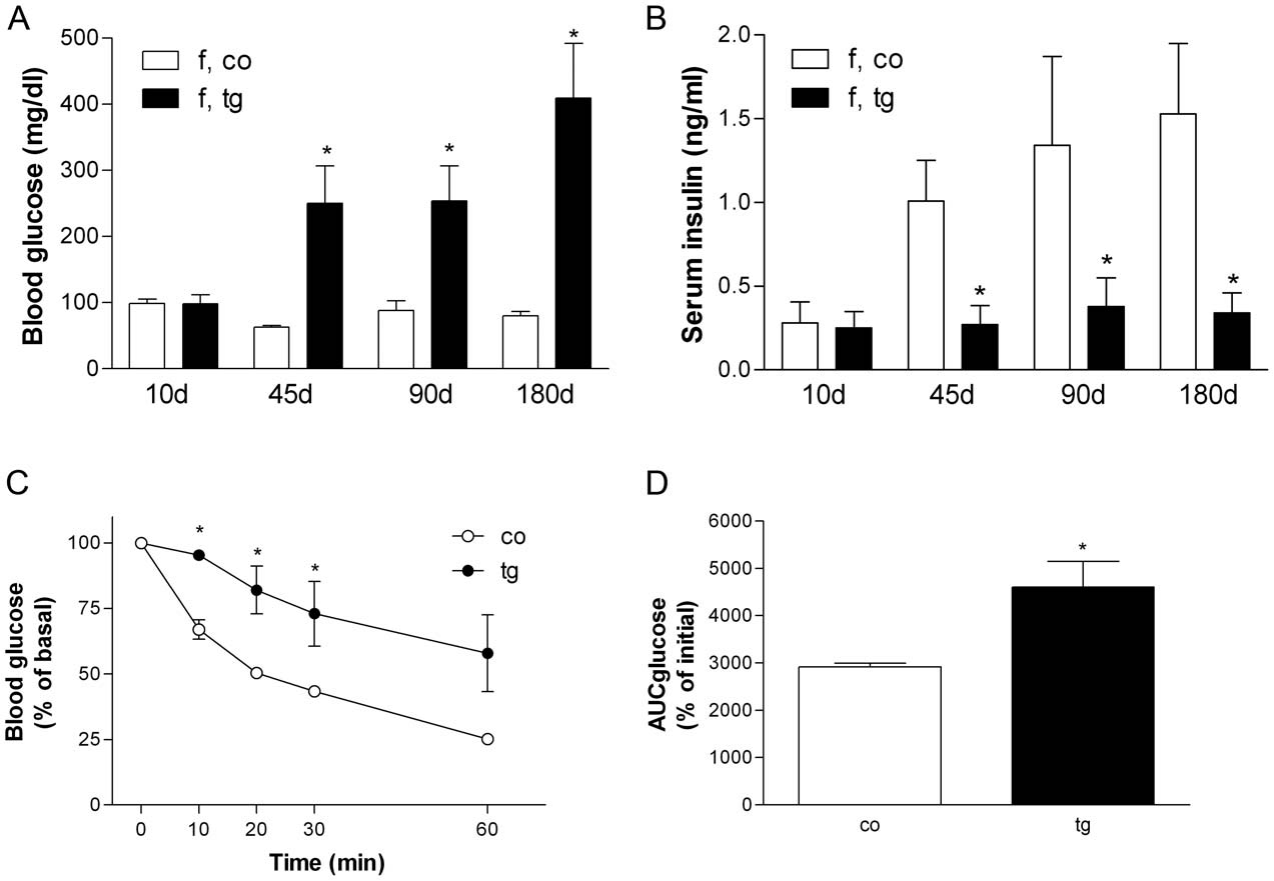
In vivo investigations. (A) Randomly fed (10 days) and fasting blood glucose (45-180 days), and (B) randomly fed (10 days) and postprandial serum insulin levels (45–90 days), (C) insulin tolerance test, (D), and area under glucose curve (AUC) during insulin tolerance test shown in C; co, control; tg, GIPR^dn^ transgenic; Data represent means and SEM, * p<0.05 vs. age-matched control.

At ten days of age, there was no difference in blood glucose or serum insulin levels between transgenic and control mice. At 45, 90 and 180 days of age, fasting blood glucose levels were significantly higher in transgenic mice (Fig. 4A) and serum insulin levels were about 50% lower in transgenic mice vs. controls (Fig. 4B). Serum malondialdehyde (MDA) levels were comparable in 45-day-old mice, and were significantly increased in 90- and 180-day-old GIPR^dn^ transgenic mice vs. controls (Table 1).

**Table 1:** Lipid peroxidation.

	MDA (µmol/l)		
	45 d	90 d^++^	180 d^++^
Co	48±18	40±11^++^	41±6^++^
Tg	35±9	79±12* ^+^	296±24* ^+^

Serum malondialdehyde (MDA) levels of GIPR^dn^ transgenic (tg) and control (co) mice at 45, 90 and 180 days of age. Data represent means and SEM;

*p<0.05 vs. age-matched control;

+ p<0.05 vs. previous time point.

Glucose disposal in response to exogenous insulin was significantly reduced in transgenic mice, 10, 20 and 30 minutes after insulin administration (Fig. 4C), and the area under glucose curve was significantly higher in GIPR^dn^ transgenic mice vs. controls (Fig. 4D).

### Preserved incretin effect on insulin secretion in 10-day-old GIPR^dn^ transgenic mice

**Figure 5:**
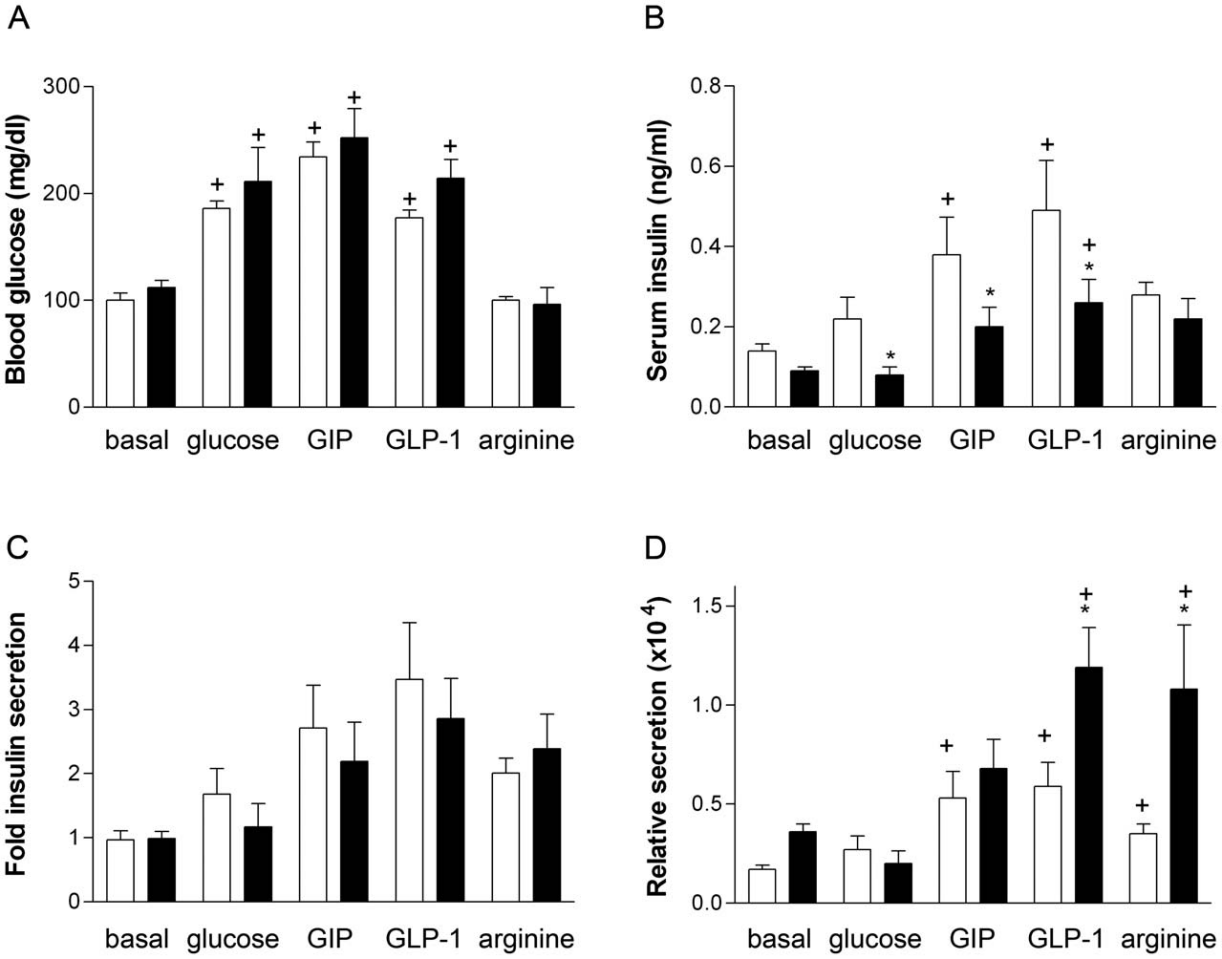
In vivo insulin secretion studies at 10 days of age. (A) Blood glucose, (B) serum insulin levels, (C) increase of serum insulin levels from basal levels (fold insulin secretion), (D) serum insulin to pancreatic insulin ratio; Open bars: control mice; filled bars: GIPR^dn^ transgenic mice; Data represent means and SEM. * p<0.05 vs. age-matched control; + p<0.05 vs. basal values.

Glucose administration alone or with GIP or GLP-1 lead to a significant increase in blood glucose levels vs. basal levels in all animals investigated (Fig. 5A). GIPR^dn^ transgenic mice showed a significantly lower (∼70%) pancreatic insulin content vs. controls (2439±641 ng/ml vs. 8027±2306 ng/ml), and serum insulin levels after stimulation with glucose, GLP-1 and GIP were significantly lower than those of control mice, whereas basal and arginine stimulated insulin levels did not differ from controls (Fig. 5B). In controls, GIP- and GLP-1-stimulated serum insulin levels were significantly higher, arginine stimulated insulin levels were by trend higher (p = 0.062) than basal levels. In transgenic mice, GLP-1-stimulated insulin concentrations were also significantly higher than basal levels (Fig 5B). However, there were no differences in serum insulin relative to basal levels (fold insulin secretion) comparing GIPR^dn^ transgenic and control mice, irrespective of the secretagogue applied (Fig. 5C). In addition, the serum insulin to pancreatic insulin ratio (relative secretion) was significantly higher following GIP, GLP-1 and arginine stimulation vs. basal ratio in control mice. In transgenic mice, relative secretion following GLP-1 and arginine was significantly higher, and following GIP stimulations was by trend higher (p = 0.07) than basal ratios. GLP-1 and arginine induced serum insulin to pancreatic insulin ratios were significantly higher in transgenic mice vs. controls (Fig 5D).

### Postnatal development of islets and beta-cells of GIPR^dn^ transgenic mice

Transgenic mice exhibited significantly lower total islet and beta-cell volumes than control mice, irrespective of the age at sampling (Fig. 2A, B). Accordingly, the total numbers of islets and beta-cells were significantly lower in transgenic mice at all time points investigated (Fig. 2C, E). The mean islet and beta-cell volumes were equal in transgenic and control mice at 10 and 45 days of age, but were significantly smaller at 90 and 180 days of age in transgenic mice vs. controls (Fig. 2D, F). In contrast to controls, total beta-cell volumes and numbers decreased significantly between 45 and 90 days of age (Fig. 2B, E), however, the number of islets almost doubled in adult GIPR^dn^ transgenic mice between 45 and 90 days (p<0.05, Fig. 2C). Consequently, the mean islet volume initially increased but dropped by ∼70% between 45 and 90 days of age (Fig. 2D). Like in controls, the mean beta-cell volume of transgenic mice decreased significantly between 10 and 45 days of age, and remained stable thereafter (Fig. 2F).

The amount of replicating islet-cells was comparable to control mice at 10 days, and was 3-fold higher vs. controls at 45 days. At 90 days of age, no replicating islet cells were observed in GIPR^dn^ transgenic mice, which is in contrast to controls (Fig. 3A). The lack of islet and beta-cell mass expansion of transgenic mice was associated with significantly disturbed islet neogenesis vs. controls (Fig. 3B). The frequency of apoptotic islet-cells appeared slightly higher in 10- and 45-day-old transgenic mice and was significantly higher at 90 days vs. controls. At 180 days of age, no apoptotic islet cells were evident in transgenic and control mice (Fig. 3C).

### cAMP in isolated islets

The cAMP content in isolated islet of 10-day-old control and GIPR^dn^ transgenic mice did not differ (0.09±0.02 pmol/islet vs. 0.10±0.02 pmol/islet).

## Discussion

The knowledge of postnatal development of the islets of Langerhans is a crucial prerequisite for the interpretation of the changes occurring in diabetes and may have an important impact on the development of new therapeutic strategies, addressing expansion of beta-cell mass. Rodent models are primarily used to address the efficacy and safety of novel therapies in preclinical studies. Several excellent studies on mechanisms of postnatal islet and beta-cell growth have been published in the past, including studies addressing islet- and beta-cell mass expansion and different lineage-tracing studies [1], [20], [21]. Detailed analyses of the age-related development of the numbers and mean volumes of islets and beta-cells have been performed in rats, investigations in mice however are scarce [22], [23], [24]. Therefore, we chose to investigate postnatal development of the endocrine pancreas in mice, using state-of-the-art stereological methods [12], [14], [15], [25], [26].

### Age-related changes in islet and beta-cell mass of CD1 mice

In healthy CD1 mice, both islet and beta-cell mass increased significantly until 90 days of age and stayed constant thereafter. These findings are in line with our previous analyses and observations in other studies on rodents [12], [22], [27], [28]. Similar changes were suggested to occur in humans, since the stimulated C-peptide response increases from childhood to adolescence in individuals at risk for type 1 diabetes mellitus [13]. It was shown that beta-cell mass in non-diabetic humans also increases with age, the largest growth rates being observed within the first six years of life [29]. We could show in the present study that the early expansion of total islet and beta-cell volumes of control mice was associated with a high rate of both islet-cell replication and neogenesis of islets. The observed high replication rate supports the concept that postnatal expansion of islet or beta-cell mass is due to replication of existing islet-cells, rather than neogenesis from precursors or stem cells [2], [4], [30], [31]. However, neogenesis has recently been shown to play an important role in regeneration of the endocrine pancreas after partial duct ligation [32].

High replication rates of islet-cells were reported between one and two, and between three and four months of age in rodents, the time course when total beta-cell mass doubled in healthy rodents. Thereafter, replication of islet cells was rather slow and the rate of replication reached that of apoptosis (2–3% per day), leading to a stable beta-cell mass [27]. In humans, a wave of beta-cell apoptosis occurs perinatally [33], replication rates are highest at gestation and in young children under about 6 months of age, and decline to low levels thereafter [29], [33].

### Age-related changes in islet and beta-cell size and number of CD1 mice

The present study shows that the postnatal increase of islet and beta-cell mass of healthy female CD1 mice is associated with a pronounced increase in number of islets and beta-cells (i.e. hyperplasia of islets and beta-cells), whereas the mean islet size does not change (no islet hypertrophy). Further, the mean beta-cell size decreased significantly between 10 and 45 days of age in control mice. The above findings are in contrast to some other observations in rodents, which have suggested that the islet number is fixed in the early postnatal period, and that the growth of the endocrine pancreas is due to islet hypertrophy [1], [2], [3], [4], [34]. The same conclusion was made in a recent study from humans [29]. However, in one study, examining the postnatal development of islet number and size in the rat, using unbiased model-independent quantitative-stereological techniques, the authors show that islet number increases significantly with age, while the mean size of the islets even decreases [23]. In male BALB/c mice between 1 week and 10 months of age, islet numbers were recently quantified after collagenase digestion of the pancreas, showing that the number of islets that may be harvested, increased largely in the first two months of life and stayed stable thereafter [24]. We showed that islet number increases significantly up to 3 months of age in female CD1 control mice, and a trend of a further increase in islet number up to 6 months was observed. Another study in rats showed that the mean beta-cell profile area does not change in rats from 2 to 31 days of age, whereas the beta-cell number triples [1], [22]. Likewise, another report demonstrated that mean beta-cell volumes of rats remained constant from 21 days of age, onwards [27]. These differences to our investigations in CD1 mice could be strain and gender specific or result from different methodologies used for analyzing islet and beta-cell size and number.

In spite of high islet-cell replication and a low rate of apoptosis, mean islet size did not change with age in female CD1 mice examined in the present study. Therefore, fission of islets might contribute to the increase in islet number. It has been suggested that fission of islets, or budding of new islets from existing ones, occurs in mice and rats, in order to keep an optimal islet size [2], [6], [16], [23]. Another explanation for a constant mean islet size of female CD1 controls could be the neonatal wave of apoptosis, which is thought to be highest in the first weeks of life when a remodeling of the endocrine pancreas takes place [22]. In the present study we could not demonstrate higher levels of apoptosis in sibling CD1 mice as compared to mice after weaning, however, since morphological signs of apoptosis are short-lived, and apoptotic cells are rapidly cleared by macrophages, the exact rate of islet-cell apoptosis is difficult to determine [28], [35], [36]. In addition, quantification of islet-cell apoptosis in histological sections only gives a snapshot of the situation and not a long-term read out. However, different methodologies used for visualization of apoptotic cells may also account the discrepancies between our results and other investigations.

### Glucose homeostasis of GIPR^dn^ transgenic female mice

GIPR^dn^ transgenic mice investigated in the present study exhibited a diabetic phenotype after weaning, characterized by fasting hyperglycemia and hypoinsulinemia, which is in line with our previous investigations [12], [37]. Serum malondialdehyde levels were determined as marker for oxidative stress. GIPR^dn^ transgenic mice exhibited significantly increased serum malondialdehyde levels from 90 days of age onwards, indicating oxidative stress due to chronic hyperglycemia.

It was previously shown that insulin secretion following a glucose challenge was largely reduced in diabetic GIPR^dn^ transgenic mice and could not be augmented by administration of GLP-1, suggesting the loss of functional beta-cell mass and/or non-specific effects of transgene expression [12]. In the present study, insulin secretion following stimulation with glucose, glucose with GIP or GLP-1 and arginine was determined before the onset of hyperglycemia in 10-day-old animals. There were no significant differences in basal and arginine stimulated serum insulin levels comparing transgenic and control mice. In GIPR^dn^ transgenic mice, glucose, GIP and GLP-1 stimulated insulin levels were significantly lower than those of control mice; however, since the pancreatic insulin content and beta-cell numbers were already reduced by 70% in transgenic animals, the reduced serum insulin levels may be a consequence of reduced beta-cell mass, rather than due to a secretion defect. The increase of insulin levels from basal (Fig. 5C) did not differ between the genetic groups, irrespective of the secretagogue applied, arguing against non-specific secretion defects due to e.g. recruiting of intracellular signaling molecules by overexpression of the dominant negative receptor. In addition, the serum insulin to pancreatic insulin ratio was calculated, showing that the remaining beta-cells of GIPR^dn^ transgenic mice respond normally to pharmacological doses of GIP. This is in line with data obtained from 11-week-old GIPR^dn^ transgenic pigs 10 minutes after application of glucose and GIP [38]. In contrast to mice lacking the GIPR that do not respond to GIP, GIPR^dn^ transgenic mice and pigs exhibit functional endogenous GIP receptors that compete with the GIPR^dn^ for the ligand GIP, explaining why GIPR^dn^ transgenic animals may respond to GIP stimulation. GIPR^dn^ transgenic mice even show two-fold higher serum insulin to pancreatic insulin ratios vs. control mice following GLP-1 or arginine stimulation. Likewise, GIPR^-/-^ mice showed an upregulation of the GLP-1 component of the enteroinsular axis [39], and GIPR^dn^ transgenic pigs showed increased responsiveness to exendin-4 [40]. Studies in GIPR and GLP-1R knockout mice demonstrated increased cAMP levels following GLP-1 or GIP stimulation, respectively. Unfortunately, isolated islets of GIPR^dn^ transgenic mice are too fragile to use them for *in vitro* stimulation assays and do not survive over night recovery after isolation procedure, therefore we could only determine basal cAMP in islets right after isolation, which did not differ between the genetic groups. Likewise, basal cAMP was not different comparing GIPR^-/-^ and wild-type mice [41]. Taken together, the results from the *in vivo* secretion studies argue against non-specific effects of transgene overexpression on insulin secretion.

In addition we have shown in the present study that, GIPR^dn^ transgenic mice exhibit slight insulin resistance. Insulin resistance has been observed in other mouse models with insulin deficient diabetes [14] and results from chronically elevated blood glucose levels [42], and reduced fat and muscular tissue [37].

### Age-related changes of the endocrine pancreas in female GIPR^dn^ transgenic mice

In female GIPR^dn^ transgenic mice analyzed in the present study, total volumes as well as absolute numbers of islets and beta-cells were significantly reduced vs. age-matched control mice (hypoplasia of islets and beta-cells). In human type 2 diabetic patients, conflicting data exist about beta-cell mass. Several studies reported a decreased total volume or volume density of beta-cells in the pancreas of diabetic patients [41], [43], whereas others did not observe a reduction in beta-cell mass [44], [45]. Mean beta-cell volumes and beta-cell numbers of GLP-1R deficient mice were comparable to wild-type mice [46], [47]. Beta-cell mass was also unaltered in *Glp1r* knockout mice as compared to wild-type mice, however, islet regeneration following partial pancreatectomy was largely disturbed in *Glp1r* knockout mice [47]. In *Gipr* ^-/-^ mice, beta-cell area as a percentage of total pancreatic area (i.e. volume density of beta-cells in the pancreas) was significantly increased in knockout vs. wild-type mice. However, the staining intensity for insulin was reduced in *Gipr* ^-/-^ islets, and the pancreatic insulin content was significantly lower [39]. In double incretin receptor knockout mice, the beta-cell to pancreas area-ratio was also unaltered vs. wild-type mice [48]. The difference in the pancreatic phenotype of GIPR^dn^ transgenic mice vs. the knockout models could be explained by yet undefined compensatory mechanisms of incretin receptor knockout models that are not triggered in GIPR^dn^ transgenic mice (or pigs, [38]), that harbor functional endogenous GIP receptors. Therefore we hypothesize that early disruption of GIPR-signaling, induced by a competition of the mutated and the endogenous wild-type receptor for the ligand GIP [12], primarily induces a disturbance in the expansion of the endocrine pancreas. However, it cannot be completely excluded that the mutant GIP receptor might recruit intracellular G-proteins, leading to reduced signaling of other G-protein coupled receptors of the beta-cells (GLP-1, glucagon receptor). To our knowledge, GIPR mutations have only been investigated in type 2 diabetic patients but not in patients exhibiting neonatal or maturity onset diabetes mellitus of unknown cause. Taking the changes in beta-cell mass of GIPR^dn^ transgenic mice and pigs [38] into account, screening for GIPR mutations in human subjects other than type 2 diabetics may be indicated.

Islet neogenesis, as evaluated by the total volume of isolated beta-cells, was severely reduced in GIPR^dn^ transgenic mice vs. controls, which is in line with our previous investigations [12], and islet-cell replication was significantly lower vs. controls, implying disturbed development of the endocrine pancreas due to a decrease in replication of existing beta-cells and reduced neogenesis from endocrine progenitors [40], [49]. GIP is known to influence beta-cell apoptosis and survival via cAMP-dependent modulation of ERK1/2 and p38MAPK [9], [50]. Islet-cell apoptosis in pancreas sections was only by trend increased in 10-day-old controls and GIPR^dn^ transgenic mice. Therefore, disturbed islet neogenesis initially appears to mainly account for the reduction in beta-cell mass of GIPR^dn^ transgenic mice, arguing against massive toxic effects of transgene expression on islet-cell survival [51]. However, since both GIP and GLP-1 are known to exert proliferative, and anti-apoptotic effects on beta-cells *in vitro*, and *in vivo* [52], [53], an increased frequency of apoptotic islet-cells would be expected in GIPR^dn^ transgenic mice. Apoptotic cells are rapidly cleared by macrophages therefore, the determination of the exact frequency of apoptotic cells in histological sections is difficult and may lead to the large variation in this parameter.

During postnatal pancreas development, the number of islets increased significantly between 45 and 90 days of age in GIPR^dn^ transgenic mice. The total volume of isolated beta-cells was highest in 10-day-old transgenic mice and significantly declined thereafter. Therefore, islet neogenesis does not appear to be completely abolished in GIPR^dn^ transgenic mice. However, the ∼2-fold increase in islet number suggests that fission of islets occurs, similar to controls [6], [23]. Unlike in controls, the mean islet size initially increased in transgenic mice. Therefore, high replication would be expected, in order to increase islet size, and actually we demonstrate here that the mean frequency of replicating islet-cells was three-times that of controls at 45 days of age. The expansion of beta-cell mass in response to glucose is generally believed to be associated with an increase of not only beta-cell number but also mean size of beta-cells [35], [54], [55], [56], [57]. Therefore we suggest that acute/sub-acute hyperglycemia could account for the high frequency of replicating islet-cells observed in GIPR^dn^ transgenic mice at 45 days of age [58], [59].

In contrast to the younger age-groups, apoptosis was significantly increased and islet-cell replication was lacking in transgenic mice vs. controls at 90 days, resulting in a significant decline of the total beta-cell volume, beta-cell number, and mean islet size with age. The observations of the present study imply progressive beta-cell loss occurring around 90 days of age, due to chronic hyperglycemia (glucotoxicity) [2], [59]. Serum malondialdehyde levels, a marker for oxidative stress, were significantly increased in transgenic mice from 90 days of age onwards vs. controls, supporting the hypothesis of beta-cell loss due to glucotoxicity. Glucose has been reported to have an ambiguous effect on beta-cell mass. Glucose in limited concentrations administered for a short period of time leads to an increase in beta-cell mass, while excessive amounts of glucose over a prolonged period exert negative effects on beta-cell function and also may negatively affect beta-cell mass by inducing apoptosis [59]. Beta-cell hypertrophy is reported in studies using glucose infusions to stimulate expansion of beta-cell mass of non-diabetic and mildly diabetic rats [60]. Beta-cell hypertrophy may also contribute to the increase in beta-cell mass as a means of adaptation to an increased insulin demand due to insulin resistance in diabetic subjects [61], [62]. In contrast, GIPR^dn^ transgenic mice investigated in the present study not only show reduced numbers, but also reduced mean volumes of beta-cells. Similarly, no compensatory beta-cell hypertrophy was observed in type 2 diabetic patients despite chronic exposure to high glucose levels [63].

In summary, we could show that physiological postnatal growth processes of the endocrine pancreas of female CD1 mice involve islet and beta-cell hyperplasia, but no considerable islet or beta-cell hypertrophy. Replication of islet-cells, neogenesis and fission of islets were demonstrated to contribute to postnatal expansion of the murine endocrine pancreas. GIPR^dn^ transgenic mice exhibit disturbed islet neogenesis, but fission of islets, and islet-cell apoptosis are initially unaltered. The resulting early reduction in beta-cell number leads to the severe diabetic phenotype of GIPR^dn^ transgenic mice after weaning, with a further age-related decline of beta-cell numbers, the latter being most likely attributable to oxidative stress.
